# Algae Incorporation and Nutritional Improvement: The Case of a Whole-Wheat Pasta

**DOI:** 10.3390/foods12163039

**Published:** 2023-08-13

**Authors:** Bárbara C. C. Oliveira, Marlene Machado, Susana Machado, Anabela S. G. Costa, Sílvia Bessada, Rita C. Alves, Maria Beatriz P. P. Oliveira

**Affiliations:** REQUIMTE/LAQV, Department of Chemical Sciences, Faculty of Pharmacy, University of Porto, Rua Jorge Viterbo Ferreira, 228, 4050-313 Porto, Portugal

**Keywords:** *Spirulina*, *Himanthalia elongata*, amino acid profile, functional food, antioxidant activity

## Abstract

Algae contain high-quality proteins, dietary fiber, minerals, and phenolic compounds, making them promising alternative ingredients. Since pasta is consumed worldwide, it can be an effective vehicle for incorporating algae. This study compares the nutritional and antioxidant composition of whole-wheat pasta without and with enrichment of an algae mixture (containing *Himanthalia elongata* and *Spirulina*) and ascertains the influence of the cooking procedure on their features. *Spirulina* and *H. elongata* were also analyzed in parallel for comparison purposes. Macronutrients, chlorides and salt, total and free amino acid profiles, and antioxidant properties (total phenolic content and ferric reducing antioxidant power) were analyzed using AOAC, Mohr’s, high performance liquid chromatography with fluorescence detection, and spectrophotometric methods, respectively. The results show a significant increase in fat (70.4%), protein (29.7%), ash (26.5%), and total amino acid (except for serine, tryptophan, isoleucine, and threonine) contents in the raw algae-enriched pasta. The antioxidant activity was also higher (4.15 versus 3.68 g ferrous sulfate eq./g dw, respectively). After cooking, protein, dietary fiber, total amino acids (except threonine) and antioxidant activity were stable in the algae-enriched pasta. Thus, algae can be an excellent ingredient for food applications with health benefits.

## 1. Introduction

Considering the growing world population, sustainable and innovative food systems are required to feed an expected 9.7 billion people by 2050 [1]. According to Geyik et al. [2], 60% of the global population lives in countries at risk of hidden hunger (micronutrient deficiency). Food production should not only aim to feed people by supplying sufficient calories, but also to nourish them by giving essential vitamins and minerals (micronutrients) [2]. Currently, food production systems face serious problems such as freshwater scarcity, damaged and infertile land, and pesticide overuse [1,3]. Algae (microalgae and macroalgae) may be a promising solution to overcome all these challenges. 

However, some direct and indirect environmental impacts should be considered in algae production. Direct impacts may include spread of non-indigenous species, loss of native biodiversity, shading and smothering of reef-building corals, and degradation of plastic ropes in the sea [4,5]. After seaweed cultivation, one should consider the upstream processes (drying, transport, and processing) that cause indirect environmental impacts. Biomass drying is the process with the highest environmental impact, so low-energy strategies involving energy available at sea (wind, waves, or currents) should be considered. Thus, the design of algae cultivation systems as well as life cycle analysis become essential in mitigating the potential negative environmental effects of algae production. Nowadays, companies concerned with environmental, food, and ecological sustainability adopt measures such as drying at low temperatures to preserve maximum nutritional value, recollecting native species, and hand-collecting algae to select algae at the optimum point of development before they wither [6].

It is also worth considering that algae cultivation can contribute to the bioremediation of nitrogen and phosphorus in estuaries or coastal areas near agricultural regions, or in integrated multi-trophic aquaculture to mitigate the impacts of fish farming [5].

Microalgae (unicellular photosynthetic microorganisms) and macroalgae (macroscopic pluricellular photosynthetic organisms) exhibit a high growth rate, cultivation on non-arable land, growth in non-potable water (seawater), and survivability in adverse conditions [6,7,8]. In addition, algae are an excellent source of nutrients, such as dietary fiber (up to 76% dw), protein (up to 70% dw), lipids (up to 50%), mainly polyunsaturated fatty acids (eicosapentaenoic acid and docosahexaenoic acid), minerals (up to 36% dw) (P, Mg, K, Ca, Fe, Zn, I), and vitamins (E, B_1_, B_2_, B_3_, B_9_) [9,10,11,12,13]. Other non-nutritive compounds with health effects can also be found in algae such as phenolic compounds (phenolic acids, flavonoids, and tannins) and carotenoids (fucoxanthin, astaxanthin) [11,14,15]. The chemical composition of algae changes depending on the species, maturity, exposure to light intensity, salinity, nutrient availability, and ecology of the environment [11,14]. Due to the presence of bioactive compounds, the integration of algae into the human diet can provide cardioprotective, antioxidant, anticancer, chemoprotective, and immunomodulatory activity [5,11,16]. Dietary fiber present in macroalgae can positively modulate the gut microbiota, increasing the levels of beneficial bacteria (*Lactobacillus* and *Bifidobacterium*) [17].

Incorporating algal or whole algal ingredients into food products can improve nutritional, technological, and bioactive properties [8,18]. In recent years, studies have described a variety of food products formulated with biomass/extracts of macroalgae or microalgae [19,20,21,22,23,24,25,26].

*Spirulina* (*Arthrospira*) is one of the most commonly incorporated microalgae in pasta, with technological (firmness, color), nutritional (protein, amino acids, iron, calcium, and magnesium), and functional (total phenolics, flavonoids, chlorophylls, and carotenoids) improvements [19,20,21,22,23,24]. Indeed, *Spirulina* has a high protein content (60–77% dry weight) and also high digestibility due to the nature of the cell wall, which is rich in digestible polysaccharides [19,21,25]. However, *Spirulina* has a negative effect on the sensory characteristics of pasta, presenting a musty–earthy flavor, and is accepted only in small amounts (up to 7%) [19,26]. According to a recent study, adding lemon–basil flavor to *Spirulina*-enriched pasta can help mask the distinctive taste of these microalgae [26].

To our knowledge, there is no research including the macroalgae *Himanthalia elongata* in pasta. However, some studies have incorporated *H. elongata*, also known as sea spaghetti, into meat products as a salt substitute. By integrating *H. elongata* into sausages, restructured meats, and meat emulsions, a 50–75% reduction in salt can be achieved compared to standard recipes [27]. Furthermore, food products developed with *H. elongata* are associated with decreased cooking loss and lipid oxidation, as well as increased phenolic content and antioxidant activity [28,29].

The aim of this study was to compare the nutritional features, including the amino acid profiles and the antioxidant potential, of whole-wheat pasta with and without enrichment (using a mixture of 2% *H. elongata* and 1.5% *Spirulina*). To ascertain the influence of the cooking procedure on nutritional and antioxidant properties, pasta samples were analyzed raw and after being cooked according to the manufacturers’ recommendations. Moreover, *Spirulina* and *H. elongata* dry samples were also analyzed in an attempt to understand if the obtained results are a consequence of the algae enrichment.

## 2. Materials and Methods

### 2.1. Reagents and Standards

Sulfuric acid (96%), petroleum ether, methanol, silver nitrate solution 0.1 M, and hydrochloric acid ≥ 37% were from Honeywell Fluka (Düsseldorf, Germany). Kjeldahl tablets and di-sodium hydrogen phosphate were from EMD Millipore Corporation (Darmstadt, Germany). Sodium dihydrogen phosphate was from VWR Chemicals (Leuven, Belgium). Carlo Erba Reagents (Val de Reuil, France) supplied hydrochloric acid (1 M) and sodium hydroxide (1 M). Ethanol (96%) was from AGA (Prior Velho, Portugal) and acetone from Fischer Chemicals (Leicestershire, U.K). Boric acid and acetic acid (99–100%) were from Chem-Lab (Zedelgem, Belgium). Sigma-Aldrich (St. Louis, MI, USA) provided sodium carbonate, gallic acid, sodium nitrate, aluminum chloride, sodium acetate, ferric chloride, TPTZ 10 mM, ferrous sulphate, a total dietary fiber assay kit, celite, and catechin. The determined amino acids have different purity grades (from ≥99.5% to 98.0%) and they were provided by Sigma-Aldrich (Buchs, Switzerland). Sigma (Deisenhofen, Germany) provided L-Norvaline. Borate buffer (0.4 N, pH 10.2), o-phthalaldehyde/3-mercaptopropionic acid (OPA/3-MPA), and 9-fluorenylmethyl chloroformate (FMOC) were from Agilent Technologies (Palo Alto, CA, USA). Disodium tetraborate decahydrate 99–103%, di-sodium hydrogen phosphate anhydrous for analysis (≥99.0%), potassium hydroxide, anhydrous sodium sulphate, Folin–Ciocalteu (FC) reagent, and potassium chromate 10% were from Merck (Darmstadt, Germany). Sodium azide (99%), HPLC-grade methanol and acetonitrile were from Honeywell Riedel-de Haën ^TM^ (Seetze, Germany). All other reagents were of analytical grade. A Milli-Q water purification system (Millipore, Bedford, MA, USA) supplied the ultrapure water used.

### 2.2. Samples and Sample Preparation

Two whole-wheat pastas were analyzed: a typical one, composed of whole durum wheat semolina only, and an algae-enriched one, composed of whole durum wheat semolina containing 2% *H. elongata* and 1.5% *Spirulina* (kindly provided by ALGAMAR, Pontevedra, Spain). The samples were subjected to a cooking procedure and analyzed both raw and after the thermal processing to ascertain any influence on the nutritional or antioxidant composition. In brief, 250 g of each sample was cooked for 15 min in 700 mL of water and 5 g of salt, according to the manufacturers’ recommendation. After cooking, samples were drained, freeze-dried, milled, and adequately stored. Dry *H. elongata* and *Spirulina* were also provided by the same company and analyzed for comparison and clarification of the enrichment process. *H. elongata* was milled at the laboratory, while *Spirulina* was already in powder form.

### 2.3. Nutritional Analysis

Several parameters were determined according to AOAC procedures [30]: ash after incineration at 500 ºC (AOAC 920.153); total fat through the Soxhlet method, in continuous extraction (AOAC 991.36); total, soluble, and insoluble fiber (TDF, SDF, and IDF) through the enzymatic–gravimetric method (AOAC 985.29) using the Total Dietary Fiber Assay Kit (TDF 100A-1KT); protein through the Kjeldahl method (AOAC 928.08). The nitrogen conversion factors 5.83 and 5 were used to calculate the total protein content for all pasta samples and for algae samples, respectively, according to FAO recommendations [31]. Moisture content was determined directly using an infrared balance (Scaltec model SMO01, Scaltec Instruments, Heiligenstadt, Germany) and available carbohydrates obtained, by difference, according to the following equation: available carbohydrates = 100% − (% moisture + % protein + % fat + % ash + % TDF). Chlorides and salt were determined using Mohr’s method. Briefly, 5 g of each sample was incinerated at 500 °C until ashes were obtained. Then, 100 mL of deionized water was added to the ashes and the solution filtered. Next, 10 mL of this solution, transferred to an Erlenmeyer flask, was titrated with silver nitrate 0.1 M in the presence of potassium chromate (10%) as an indicator. A red color shows the endpoint of the titration.

### 2.4. Amino Acid Profile Analysis

#### 2.4.1. Free Amino Acid Extraction

Free amino acid profiles were determined according to the method of Machado et al. [32] with slight modifications. Briefly, 10 mL of deionized water and 1 g of sample were stirred for 30 min at 40 °C for the amino acid extraction, followed by centrifugation. The supernatant was collected and the residue re-extracted with 5 mL of deionized water for 15 min, then it was centrifuged and the supernatants were combined. Finally, after homogenization, the supernatant was centrifuged. Then, 990 µL was mixed with 10 µL of the internal standard (norvaline, 2 mg/mL).

#### 2.4.2. Total Amino Acid Extraction

The total amino acid profile was determined according to the method of Pimentel et al. [33] and Machado et al. [32]. Briefly, 150 mg of sample and 3 mL of 6 M HCl were mixed in a tube and subjected to a N_2_ stream to minimize oxidation. Immediately, the tubes were capped and heated at 110 °C for 24 h in a thermoblock (SBH130D/3, Stuart, Stafford, UK). The hydrolyzed samples were further centrifuged. Next, 50 µL of the supernatant was collected and neutralized with 940 µL of borate buffer (pH 10.2), and the internal standard (10 µL norvaline, 2 mg/mL) added. The mix was centrifuged, and supernatants were finally transferred into injection vials for analysis.

An alkaline hydrolysis was also performed to determine tryptophan contents, as this amino acid degrades under acidic conditions, and a similar protocol of acid hydrolysis was used. In brief, 150 mg of each sample was mixed with 3 mL of 4 M KOH in a tube. The tubes were capped and heated in a thermoblock at 110 °C for 4 h. After hydrolysis, the samples were neutralized with 0.1 M HCl.

#### 2.4.3. Chromatographic Separation and Detection

The samples were automatically derivatized in an autosampler as described by Machado et al. [32]. Two derivatization reagents were used: OPA, which, in the presence of a thiol compound (3-MPA), reacts with primary amines to produce highly fluorescent isoindole products and FMOC to derivatize secondary amines. Amino acids were analyzed using reversed-phase high performance liquid chromatography with fluorescence detection and the peak identification was based on the retention time of the corresponding standards. The quantification of the amino acids was performed using the internal standard method.

### 2.5. Total Phenolic Content and In Vitro Antioxidant Activity

#### 2.5.1. Extract Preparation

Pasta extracts were prepared using 2.5 g of sample and 25 mL of solvent (50% ethanol/50% water; *v*/*v*) on a heating plate at 40 ± 2 °C with constant stirring for 15 min. Extracts of *Spirulina* and *H. elongata* were also prepared, using 200 mg of each in 25 mL of the same solvent. All extracts were prepared in triplicate, filtered (paper filter Whatman No. 4), and stored at −20 °C for further analyses.

#### 2.5.2. Total Phenolics Content (TPC)

The amount of total phenolics was determined according to the method of Costa et al. [34]. TPC of extracts were determined through spectrophotometric measurement using a calibration curve with increasing concentrations of a standard solution of gallic acid (5–100 mg/mL; R^2^ = 0.9997). First, 30 µL of each extract was mixed with 150 µL of FC reagent and 120 µL of sodium carbonate (7.5%; m/v), incubated at 45 °C for 15 min, and then cooled down to room temperature for 30 min, always protected from light. The absorbance was measured at 765 nm and the results are expressed as gallic acid equivalents (GAE).

#### 2.5.3. Ferric Reducing Antioxidant Power (FRAP)

The FRAP assay was carried out according to the method of Costa et al. [35]. A calibration curve of a standard solution of ferrous sulphate with increasing concentrations (25–500 μmol/L; R^2^ = 0.9986) was used and each extract analyzed in triplicate, in the microplate reader Synergy HT BioTek. First, 35 µL of the extract was mixed with 265 µL of FRAP reagent (0.3 M acetate buffer, 10 mM TPTZ solution, and 20 mM of ferric chloride) and incubated at 37 °C, protected from light, for 30 min. Then, absorbance was measured at 595 nm. The results are expressed as ferrous sulphate equivalents (FSE).

### 2.6. Statistical Analysis

Statistical analysis was performed using IBM SPSS v. 25 (IBM Corp., Armonk, NY, USA). Data are expressed as mean ± standard deviation. Measurements were performed in triplicate. One-way ANOVA was used to assess significant differences between samples, followed by Tukey’s HSD or Dunnett T3 post hoc test (selected based on the equality of the variances) to make pairwise comparisons between means. The level of significance for all hypothesis tests (*p*) was 0.05. Student’s *t*-tests were used to discriminate between any two groups under consideration.

## 3. Results and Discussion

In this study, the nutritional parameters of RP (whole-wheat raw pasta), CP (whole-wheat cooked pasta), RPA (whole-wheat raw pasta with algae), CPA (whole-wheat cooked pasta with algae), SPI (*Spirulina*), and HIM (*H. elongata*) were assessed. Additionally, chloride content, total phenolic content, and potential antioxidant activity were investigated. Special attention was given to the total and free amino acid profiles of the products.

### 3.1. Chemical Composition of the Algae and Pasta

Table 1 shows the chemical composition of all the samples (algae and pasta). *Spirulina* showed a higher protein content (59% dry weight (dw)), as well as a high percentage of TDF (30% dw) and ash (7% dw). *H. elongata* had a moderate protein content (8.8% dw), but it presented a higher ash content (33% dw) and TDF (47% dw). These values are in accordance with previously published studies [36,37,38,39]. Due to the high protein content of *Spirulina* and the TDF of *H. elongata*, it was expected that the incorporation of these algae into a foodstuff would improve its content of these nutrients. *Spirulina* and *H. elongata* had low fat content (1.10% and 0.14% dw, respectively), contributing to their low caloric value. Significant differences in salt content were found between the microalgae and the macroalgae (*p* < 0.05). *H. elongata* showed the highest content of chlorides (9.9% dw) and, consequently, NaCl (16.7% dw). This feature should be due to the different processing of each type of algae. *H. elongata* was collected from ocean water and sun-dried, keeping high levels of these minerals.

The pasta enriched with *Spirulina* (1.5%) and *H. elongata* (2%) presented significant differences in the nutritional features compared to the conventional whole-wheat pasta. In fact, nutritional parameters (dw, Table 1), such as protein, fat, SDF, and ash, are significantly higher in algae-enriched pasta (contents of 13.1, 0.46, 4.9, and 2.15%, respectively, vs. 10.1, 0.27, 2.5, and 1.70% in the 100% whole-wheat pasta). Proteins are particularly important since they contribute to maintaining the immune system, developing muscle, transporting molecules, and accelerating biochemical reactions [40]. Furthermore, protein, particularly gluten, exerts a crucial role in pasta formulation in terms of structure, texture, chewiness, and cooking quality. Wheat semolina hydration promotes protein hydration and unfolding, allowing for the formation of a gluten network around the starch granules. The gluten network confers viscoelastic properties to the pasta [41]. The addition of non-gluten proteins can affect the structure or texture of the pasta and gluten network. Koli et al. [21] found that incorporating increasing percentages of *Spirulina* (2–15%) into pasta decreases firmness, suggesting that *Spirulina* interferes with the cross-linking of gluten. Lemes et al. [42], on the other hand, showed that the addition of 5% *Spirulina* biomass increased the elasticity of the pasta compared to the whole standard pasta. Furthermore, Graça et al. [43] also found that the incorporation of 3% *Chlorella vulgaris* microalgae into wheat flour pasta improved rheology and viscoelastic properties, with strengthening of the gluten network.

No significant differences (in dw) were found for TDF (*p* > 0.05). IDF and available carbohydrate contents were significantly lower (dw) in the algae-enriched pasta (contents of 5.5 and 74.0%, respectively, vs. 6.7 and 78.7% in the 100% whole-wheat pasta). The nutritional improvement verified in this study, in terms of protein, lipid, and ash contents, is consistent with a previous study published by Koli et al. [21]. In their research, the incorporation of 2–15% *Spirulina* into semolina-based pasta resulted in an increase in protein, lipid, iron, and calcium contents up to 77.47, 37.60, 296.99, and 57.27% (dw), respectively [21]. Lemes et al. [42] also found that adding 10% *Spirulina platensis* to wheat flour pasta significantly increases protein, lipid, and TDF levels.

After being cooked and drained, the ready-to-eat pasta presented a moisture content around 57 and 59% for algae-enriched pasta and 100% whole-wheat pasta, respectively. Significant differences (*p* < 0.05) were found for protein, fat, available carbohydrates, and ash contents between the two ready-to-eat pastas (fw) (Table 1). In fact, moisture, available carbohydrates, and fat contents suffered a significant decrease with algae incorporation. In contrast, protein and total mineral (ash) contents were higher in algae-enriched pasta. Minerals have critical roles in the human body, from developing strong bones to nerve signal transmission. Mineral deficiency can be reduced by including mineral-rich ingredients in processed food formulations. Algae are rich in microelements like I, Fe, and Zn, whose deficiencies are associated with thyroid gland “goiter”, anemia, and high susceptibility to infections, respectively [44].

Comparing the algae-enriched pasta before and after cooking, but in dry weight, a decrease in the contents of lipids after cooking is observed. The protein content remained higher in the cooked enriched pasta (dw), indicating that proteins did not leach during cooking. The recommended daily protein intake for adults, irrespective of age, is 0.8 g per kilogram body weight [45]. An adult weighing 70 kg needs to consume 56 g of protein per day to avoid a negative nitrogen balance and subsequent gradual loss of lean mass [40]. In this way, a portion of 100 g of algae-enriched pasta (dw, cooked) contributes to 24% of the recommended daily intake of protein.

The incorporation of algae into the pasta had no significant effect on the chloride (Cl) or salt (NaCl) content (fw or dw). The analysis of chlorides in algae-enriched foods is crucial to determine whether the salt present in the algae contributed to the chloride content of the final product. In addition, it is an essential criterion for people who need to control their salt and chloride intake due to medical conditions such as kidney disease or hypertension. In terms of the nutritional value, the incorporation of 1.5% *Spirulina* and 2% *H. elongata* mainly influenced the protein and total mineral contents of the enriched pasta.

### 3.2. Total Phenolic Content and Antioxidant Activity

A significant nutritional improvement in the algae-enriched pasta was observed. Likewise, it was also important to investigate the behavior of the pasta in terms of antioxidant properties. Table 1 presents the obtained results for the TPC and FRAP assays. Considering the *Spirulina* and *H. elongata* extracts, significant differences (*p* < 0.05) were found for the TPC and FRAP values. *Spirulina* presented higher values (3.0 mg GAE/g for TPC and 2.9 mg FSE/g for FRAP) compared to *H. elongata*.

Previous studies reported a TPC for *H. elongata* ranging from 5.5 to 286 mg GAE/g [46,47,48]. The values found in this study fell outside of the previously mentioned range (1.27 mg GAE/g). However, different extractive conditions (w/v and solvent extractor type) were used, which can make comparisons difficult. Previous studies reported a TPC for *Spirulina* ranging from 2.49 to 7.91 mg GAE/g [49,50,51], with the values of this study being inside this range (3.02 mg GAE/g). There are studies reporting that brown seaweeds, such as *H. elongata*, contain more bioactive components than green or red seaweeds [52]. Sea spaghetti is a good source of bioactive compounds, particularly polyphenols, such as catechins, phlorotannins, flavonoids, flavanols, and flavanol glycosides [47]. Phlorotannins are the most-studied group of polyphenols, and their content is reported to be around 5–15% (dw) [15,47]. However, in this study, *Spirulina* had a higher TPC value than *H. elongata.* The production and diversity of phenolic compounds in algae are directly related to the taxonomic group, due to cellular mechanisms and genetic coding. However, extrinsic factors such as geolocation, season, salinity, pH, light exposure, and water nutrient composition can affect the amount of phenolic compounds in algae [53]. The *H. elongata* used in this study was grown on the coast of Galicia under uncontrolled conditions and therefore was subjected to stress and nutrient variation that may affect metabolic activity. In turn, the *Spirulina* used in this study was from organic production, where abiotic factors were controlled. The difference in TPC between the algae studied may also be related to the absence of cellulosic walls in *Spirulina*, which facilitates light penetration, important for photosynthesis and the production of bioactive compounds. In addition, the production system may also influence TPC in algae. For example, photobioreactors, often used in microalgae production, provide controlled and contamination-free culture conditions that contribute to high metabolic productivity [54].

The TPC results (dw) of the raw whole-wheat pasta without and with algae were not significantly different (*p* > 0.05). However, the FRAP values (dw) were significantly higher in the algae-enriched pasta (4.2 mg FSE/g against 3.7 mg FSE/g, for raw whole-wheat pasta), indicating higher antioxidant activity. After cooking, despite the lower TPC value, the algae-enriched pasta showed better antioxidant activity (although the values were not statistically different), suggesting that there might actually be a tendential improvement in the antioxidant properties due to algae incorporation. These results also suggest that other compounds besides phenolics can be responsible for the antioxidant properties. *Spirulina* is rich in photosynthetic pigments such as chlorophyll, carotenoids, and phycobiliproteins, like phycocyanin, phycoerythrin, and allophycocyanin, that are responsible for the blue-green color and constitute about 19% of its dry weight [15,38,55]. These pigments, in addition to providing color, also have antioxidant properties [55]. This may explain the higher FRAP values observed in the raw pasta enriched with algae.

Marco et al. reported a TPC of 0.6 mg GAE/g (dw, cooked) for dried wheat pasta enriched with 5% *Spirulina*, using different extraction conditions [51]. The obtained results in this study are similar (0.68 mg GAE/g dw, cooked) despite the addition of only 1.5% *Spirulina*. However, in this case, the pasta was also enriched with 2% *H. elongata,* which may influence the TPC values. To the best of our knowledge, there are no previous studies reporting the phenolics influence of this seaweed in foodstuffs.

### 3.3. Amino Acid Profile

#### 3.3.1. Total Amino Acids

Table 2 presents the total amino acid profile of all samples under evaluation. The human body does not synthesize essential amino acids, which are needed for its normal functioning. Therefore, it is vital to obtain these amino acids via dietary sources. Despite the quantitative differences among samples, all of them had a complete profile of essential amino acids.

*Spirulina* (dw) showed high amounts of all essential amino acids, especially leucine, lysine, threonine, isoleucine, and valine (51, 37, 35, 34, and 34 mg/g dw, respectively). Leucine, isoleucine, and valine are BCAA (branched-chain amino acids) with similar functions and benefits such as the synthesis of muscle proteins, increasing muscle mass, and decreasing muscle catabolism [56]. These amino acids may also have positive effects on food intake, glycemic control, and energy expenditure [57]. The contents of essential amino acids were comparable to those reported by Gutierrez-Salmean et al. [58]. Gutierrez-Salmean et al. [58] also identified leucine, valine, isoleucine, lysine, and threonine as major amino acids (53.8, 39.4, 35.0, 29.6, and 28.6 mg/g dw, respectively) and tryptophan and histidine as minor amino acids (10.9 and 10.0 mg/g dw, respectively).

The most abundant essential amino acids in *H. elongata* were histidine, leucine, and threonine (10.4, 6.6, and 5.5 mg/g dw). Histidine has anti-inflammatory properties and is essential for immune system response as a precursor of histamine [59]. In the study carried out by Biancarosa et al. [60], leucine was the most abundant amino acid, followed by lysine and valine. Tryptophan was the amino acid found in the lowest concentration in both algae (3.6 and 0.9 mg/dw for *Spirulina* and *H. elongata*, respectively).

Regarding non-essential amino acids, glutamic acid and aspartic acid were the most prevalent in *Spirulina* (86 and 64 mg/g dw, respectively) and *H. elongata* (22 and 11 mg/g dw, respectively). This result agrees with previously published studies [58,60]. In spite of being a non-essential amino acid, glutamic acid is important for the proper functioning of the brain and improves mental capacities [61]. Aspartic acid has an important role in the neuroendocrine system, as well as in the development of the nervous system [62]. The non-essential amino acid found in the lowest amount was hydroxyproline for *Spirulina* (0.13 mg/g dw) and *H. elongata* (0.30 mg/g dw). The sum of total amino acids in *Spirulina* was approximately six times greater than in *H. elongata*.

The more representative essential amino acids in pasta samples (dw) were leucine, phenylalanine, and valine. Phenylalanine acts in the formation of neurotransmitters that contribute to good brain function, mental capacity, and improved mood [63]. The amino acid found in the lowest amount was tryptophan. In comparison with the 100% whole-wheat pasta, the algae-enriched one contained significantly higher levels of the following essential amino acids: valine, methionine, phenylalanine, isoleucine, leucine, threonine, and histidine (raw or cooked, dw). In the study by Raczyk et al. [19], the addition of 3% *Spirulina* to semolina pasta increased the content of all essential and non-essential amino acids. Comparing both ready-to-eat pastas (fw), the highest increase was observed for leucine, with a difference of 0.9 mg/g, followed by valine, threonine, and isoleucine, with differences of ~0.5 mg/g each. These significant differences correspond to some of the essential amino acids present in higher amounts in both algae used (*Spirulina* and *H. elongata*).

Glutamic acid and proline were the main non-essential amino acids in all of the pasta samples (dw). The pasta also contained high levels of aspartic acid, serine, and arginine. The amino acid found in the lowest amount in pasta samples (dw) was hydroxyproline. The algae-enriched pasta had higher values of all non-essential amino acids. Comparing both ready-to-eat pastas (fw), the highest increase was for glutamic acid, with a difference of 3.1 mg/g, followed by proline, aspartic acid, and alanine, with differences of 1.1, 0.7, and 0.6 mg/g, respectively. These major differences correspond to some of the non-essential amino acids present in great quantities in both algae.

#### 3.3.2. Free Amino Acids

Free amino acids are not linked to other amino acids, peptides, or proteins and usually contribute to food taste [64]. Table 3 presents the free amino acid profile of all samples in this study. Considering essential amino acids, compared to *H. elongate, Spirulina* showed higher amounts of lysine and leucine, followed by threonine and isoleucine. In *Spirulina*, the essential amino acid found in the lowest amount was methionine (80 mg/g dw). Considering non-essential amino acids, in relation to *H. elongate, Spirulina* showed greater amounts of glutamic acid, followed by alanine and arginine.

For *H. elongata*, the free essential amino acid found in greatest amounts was histidine, and in lowest amounts was tryptophan (16 mg/g dw). Considering non-essential amino acids, compared to *Spirulina, H. elongata* showed higher amounts of free glutamic and aspartic acids and lower amounts of free hydroxyproline.

Tryptophan was the most abundant essential amino acid in the free form in pasta samples (raw and dw). For the whole-wheat pasta and algae-enriched pasta, free histidine and methionine were the least abundant essential amino acids, respectively. Non-essential free amino acids found in higher amounts in pasta samples (raw, dw) were aspartic acid for whole-wheat pasta and alanine plus asparagine for algae-enriched pasta. The non-essential free amino acid in lowest quantities was hydroxyproline for both pasta samples.

Comparing both raw pastas (dw), it is possible to see that the enrichment with algae led to an increase in several free amino acids. However, other amino acids such as aspartic acid, asparagine, methionine, and tryptophan decreased. Comparing both ready-to-eat pastas (fw), the highest increase was for histidine, with a difference of 60 µg/g, followed by alanine, threonine, leucine, and proline (algae-enriched pasta). These major differences correspond to some of the principal free amino acids present in *Spirulina* and *H. elongata*.

## 4. Conclusions

The addition of algae with distinct chemical compositions to foods seems to be an effective strategy to improve different nutritional and bioactive parameters. The enrichment of pasta with a combination of algae, namely *Spirulina* (1.5%) and *Himanthalia elongata* (2%), resulted in significant higher levels of protein, fat, soluble dietary fiber, and ash content compared to a 100% whole-wheat pasta. Furthermore, based on the total amino acid profile, the algae-enriched pasta can be considered a source of essential amino acids, especially leucine, phenylalanine, and valine. The most limiting amino acid in the algae-enriched pasta was tryptophan. In terms of bioactivity, the mix of *Spirulina* and *Himanthalia elongata* contributed to a higher antioxidant activity of the pasta without significantly affecting the content of total phenolic compounds. This study demonstrated that algae can be a valuable functional ingredient in pasta production, contributing to food security. Future studies should focus on increasing the incorporation of algae into pasta to maximize their benefits, but this should be done with a careful evaluation of technological characteristics to ensure consumer acceptability.

## Figures and Tables

**Table 1:** Nutritional analysis, total phenolic content, and potential antioxidant activity of algae and pasta.

	Nutritional Composition									
Parameters	% Dry Weight			% Dry Weight				% Fresh Weight		
	SPI	HIM		RP	RPA	CP	CPA	CP	CPA	
Moisture	-	-		-	-	-	-	58.6 ± 0.2	56.9 ± 0.3	*
Protein	59.2 ± 0.5	8.8 ± 0.3	*	10.1 ± 0.4 ^c^	13.1 ± 0.4 ^a^	11.8 ± 0.6 ^b^	13.5 ± 0.4 ^a^	6.9 ± 0.3	7.7 ± 0.2	*
Fat	1.10 ± 0.03	0.14 ± 0.00	*	0.27 ± 0.01 ^d^	0.46 ± 0.02 ^b^	0.55 ± 0.02 ^a^	0.37 ± 0.00 ^c^	0.32 ± 0.01	0.21 ± 0.00	*
TDF	29.7 ± 0.4	47.2 ± 1.8	*	9.2 ± 0.1 ^a^	10.3 ± 0.5 ^a^	10.2 ± 0.7 ^a^	10.5 ± 0.5 ^a^	6.0 ± 0.4	6.0 ± 0.3	
*IDF*	12.8 ± 0.6	18.0 ± 1.0	*	6.7 ± 0.2 ^a^	5.5 ± 0.1 ^b^	4.8 ± 0.3 ^b^	5.3 ± 0.3 ^b^	2.8 ± 0.2	3.0 ± 0.2	
*SDF*	16.9 ± 0.9	29.2 ± 0.8	*	2.5 ± 0.1 ^b^	4.9 ± 0.5 ^a^	5.0 ± 1.0 ^a^	5.2 ± 0.2 ^a^	3.2 ± 0.6	3.0 ± 0.1	
AC	2.8 ± 0.6	11.0 ± 1.1	*	78.7 ± 0.2 ^a^	74.0 ± 1.0 ^b^	75.2 ± 0.5 ^ab^	73.3 ± 0.4 ^b^	44.2 ± 0.3	41.8 ± 0.3	*
Ash	7.1 ± 0.0	32.5 ± 0.6	*	1.70 ± 0.10 ^c^	2.15 ± 0.02 ^b^	2.19 ± 0.02 ^ab^	2.37 ± 0.04 ^a^	1.28 ± 0.01	1.35 ± 0.02	*
*Cl^−^*	0.25 ± 0.04	9.9 ± 0.1	*	0.26 ± 0.04 ^a^	0.23 ± 0.00 ^a^	0.23 ± 0.00 ^a^	0.26 ± 0.04 ^a^	0.14 ± 0.00	0.15 ± 0.02	
*NaCl*	0.42 ± 0.07	16.5 ± 0.4	*	0.43 ± 0.07 ^a^	0.39 ± 0.00 ^a^	0.38 ± 0.00 ^a^	0.43 ± 0.07 ^a^	0.22 ± 0.00	0.25 ± 0.03	
	Total phenolic content and antioxidant activity									
TPC (mg GAE/g)	3.02 ± 0.40	1.27 ± 0.40	*	1.06 ± 0.05 ^a^	1.10 ± 0.20 ^a^	0.73 ± 0.03 ^b^	0.68 ± 0.04 ^b^	0.43 ± 0.02	0.39 ± 0.02	
FRAP (mg FSE/g)	2.88 ± 0.23	0.86 ± 0.20	*	3.68 ± 0.46 ^b^	4.15 ± 0.32 ^a^	4.17 ± 0.12 ^a^	4.50 ± 0.13 ^a^	2.44 ± 0.07	2.56 ± 0.07	

RP (whole-wheat raw pasta), CP (whole-wheat cooked pasta), RPA (whole-wheat raw pasta with algae), CPA (whole-wheat cooked pasta with algae), SPI (*Spirulina*), HIM (*Himanthalia elongata*), TDF (total dietary fiber), IDF (insoluble dietary fiber), SDF (soluble dietary fiber), AC (available carbohydrates), TPC (total phenolic content), GAE (gallic acid equivalents), FRAP (ferric reducing antioxidant power), FSE (ferrous sulfate equivalents). Within each line, different letters represent significant differences between RP, CP, RPA, and CPA, at *p* < 0.05. Within each line, * represent significant differences between two different samples, i.e., between CP and CPA (% fresh weight, as consumed) or between SPI and HIM, at *p* < 0.05. The results are presented as mean ± standard deviation (*n* = 3)

**Table 2:** Total amino acid composition expressed in mg/g (dw or fw) of algae and pasta.

Amino Acids	SPI	HIM		RP	CP	RPA	CPA	CP	CPA	
	Dry Weight			Dry Weight				Fresh Weight		
Non-essential										
Asp	64 ± 2	11 ± 0	*	6.7 ± 0.3 ^b^	6.5 ± 0.2 ^b^	7.6 ± 0.3 ^a^	7.9 ± 0.2 ^a^	3.8 ± 0.1	4.5 ± 0.1	*
Glu	86 ± 3	22 ± 0	*	34 ± 2 ^b^	33 ± 1 ^b^	38 ± 2 ^a^	40 ± 1 ^a^	19.6 ± 0.8	22.7 ± 0.8	*
Ser	32 ± 1	5.1 ± 0.1	*	6.4 ± 0.4 ^bc^	6.1 ± 0.2 ^c^	7.0 ± 0.3 ^ab^	7.3 ± 0.3 ^a^	3.6 ± 0.1	4.1 ± 0.2	*
Gly	32 ± 1	4.4 ± 0.1	*	4.1 ± 0.6 ^a^	3.8 ± 0.1 ^a^	4.5 ± 0.2 ^a^	4.6 ± 0.2 ^a^	2.2 ± 0.1	2.6 ± 0.1	*
Arg	50 ± 2	5.4 ± 0.2	*	5.6 ± 0.1 ^b^	5.9 ± 0. 3 ^b^	6.5 ± 0.3 ^a^	6.8 ± 0.3 ^a^	3.4 ± 0.2	3.9 ± 0.2	*
Ala	50 ± 2	7.1 ± 0.2	*	4.3 ± 0.2 ^b^	4.2 ± 0.2 ^b^	5.1 ± 0.2 ^a^	5.3 ± 0.2 ^a^	2.4 ± 0.1	3.0 ± 0.1	*
Tyr	27 ± 1	2.1 ± 0.1	*	1.8 ± 0.1 ^b^	1.9 ± 0.1 ^b^	2.4 ± 0.2 ^a^	2.5 ± 0.1 ^a^	1.13 ± 0.05	1.44 ± 0.06	*
Hyp	0.126 ± 0.005	0.30 ± 0.01	*	0.126 ± 0.007 ^a^	0.119 ± 0.003 ^a^	0.131 ± 0.006 ^a^	0.126 ± 0.004 ^a^	0.069 ± 0.002	0.072 ± 0.002	
Pro	25.1 ± 0.8	3.9 ± 0.1	*	12.0 ± 0.4 ^b^	12.1 ± 0.6 ^b^	13.6 ± 0.5 ^a^	14.3 ± 0.4 ^a^	7.1 ± 0.3	8.2 ± 0.2	*
Essential										
Val	34 ± 1	4.7 ± 0.1	*	4.6 ± 0.2 ^b^	4.6 ± 0.2 ^b^	5.3 ± 0.2 ^a^	5.6 ± 0.2 ^a^	2.7 ± 0.1	3.2 ± 0.1	*
Met	11 ± 1	2.3 ± 0.1	*	1.96 ± 0.09 ^b^	1.91 ± 0.08 ^b^	2.2 ± 0.1 ^a^	2.3 ± 0.1 ^a^	1.12 ± 0.05	1.30 ± 0.05	*
Trp	3.6 ± 0.2	0.9 ± 0.1	*	0.91 ± 0.02 ^ab^	0.99 ± 0.07 ^a^	0.79 ± 0.04 ^c^	0.88 ± 0.01 ^bc^	0.58 ± 0.04	0.500 ± 0.006	*
Phe	23 ± 1	3.4 ± 0.2	*	4.7 ± 0.1 ^b^	5.1 ± 0.2 ^b^	5.8 ± 0.3 ^a^	6.0 ± 0.2 ^a^	3.0 ± 0.1	3.4 ± 0.1	*
Ile	34 ± 2	4.4 ± 0.1	*	4.2 ± 0.3 ^bc^	3.8 ± 0.2 ^c^	4.6 ± 0.2 ^ab^	5.0 ± 0.2 ^a^	2.3 ± 0.1	2.8 ± 0.1	*
Leu	51 ± 2	6.6 ± 0.2	*	7.6 ± 0.4 ^b^	7.6 ± 0.3 ^b^	8.7 ± 0.4 ^a^	9.2 ± 0.3 ^a^	4.4 ± 0.2	5.3 ± 0.2	*
Lys	37 ± 1	4.5 ± 0.1	*	2.9 ± 0.6 ^a^	2.5 ± 0.1 ^a^	2.8 ± 0.1 ^a^	3.1 ± 0.2 ^a^	1.44 ± 0.05	1.75 ± 0.09	*
Thr	35 ± 1	5.5 ± 0.2	*	4.2 ± 0.3 ^bc^	3.8 ± 0.2 ^c^	4.4 ± 0.2 ^b^	4.7 ± 0.2 ^a^	2.2 ± 0.1	2.7 ± 0.1	*
His	15.7 ± 0.2	10.4 ± 0.2	*	3.3 ± 0.1 ^b^	3.2 ± 0.1 ^b^	3.8 ± 0.1 ^a^	3.8 ± 0.2 ^a^	1.9 ± 0.1	2.1 ± 0.1	*
Total amino acids sum (%)	61.2	10.4		10.9	10.7	12.3	12.9	6.3	7.4	

RP (whole-wheat raw pasta), CP (whole-wheat cooked pasta), RPA (whole-wheat raw pasta with algae), CPA (whole-wheat cooked pasta with algae), SPI (*Spirulina*), HIM (*Himanthalia elongata*), dw (dry weight), fw (fresh weight). Within each line, different letters represent significant differences between RP, CP, RAP, and CAP at *p* < 0.05. Within each line, * represent significant differences between two different samples (i.e., CP and CAP expressed in fw or SPI and HIM) at *p* < 0.05.

**Table 3:** Free amino acid profile expressed in µg/g (dw or fw) of algae and pasta.

Amino Acids	SPI	HIM		RP	CP	RPA	CPA	CP	CPA	
	Dry Weight			Dry Weight				Fresh Weight		
Non-essential										
Asp	325 ± 10	1171 ± 33	*	263 ± 1 ^a^	227 ± 6 ^b^	167 ± 0.8 ^c^	125 ± 1 ^d^	133 ± 3	71 ± 1	*
Glu	4323 ± 129	2671 ± 33	*	141 ± 1 ^a^	112 ± 4 ^b^	141 ± 1 ^a^	100 ± 1 ^c^	65 ± 2	57 ± 1	*
Asn	131 ± 5	89 ± 3	*	217 ± 3 ^a^	209 ± 7 ^ab^	199 ± 0 ^b^	208 ± 3 ^ab^	122 ± 4	118 ± 2	
Ser	186 ± 3	145 ± 4	*	55 ± 2 ^c^	59 ± 1 ^c^	97 ± 2 ^a^	74 ± 0 ^b^	35 ± 1	42 ± 0	*
Gln	73 ± 4	894 ± 29	*	21 ± 2 ^c^	18 ± 0 ^c^	37 ± 1 ^a^	27 ± 1 ^b^	11 ± 0	15 ± 0	*
Gly	206 ± 10	185 ± 5		40 ± 2 ^c^	41 ± 3 ^c^	67 ± 1 ^a^	59 ± 1 ^b^	24 ± 2	34 ± 1	*
Arg	338 ± 23	43 ± 2	*	89 ± 2 ^b^	94 ± 2 ^ab^	99 ± 2 ^a^	87 ± 2 ^b^	55 ± 1	49 ± 1	*
Ala	1195 ± 63	1016 ± 30	*	119 ± 3 ^c^	101 ± 2 ^d^	233 ± 2 ^a^	184 ± 4 ^b^	59 ± 1	105 ± 2	*
Tyr	262 ± 16	31 ± 1	*	43 ± 2 ^b^	38 ± 1 ^c^	53 ± 2 ^a^	39 ± 1 ^bc^	22 ± 1	22 ± 0	
Hyp	2.5 ± 0.1	3.3 ± 0.1	*	3.2 ± 0.1 ^a^	2.8 ± 0.2 ^b^	3.3 ± 0.1 ^a^	2.9 ± 0.1 ^ab^	1.6 ± 0.1	1.7 ± 1	
Pro	66 ± 0	56 ± 4		43 ± 0.1 ^c^	30 ± 1 ^d^	69 ± 3 ^a^	58 ± 2 ^b^	18 ± 1	33 ± 1	*
Essential										
His	111 ± 6	8613 ± 169	*	21 ± 1 ^c^	18 ± 2 ^c^	101 ± 3 ^b^	126 ± 2 ^a^	11 ± 1	71 ± 1	*
Thr	227 ± 7	69 ± 4	*	49 ± 1 ^c^	43 ± 0 ^d^	88 ± 1 ^a^	73 ± 2 ^b^	25 ± 0	41 ± 1	*
Val	188 ± 5	57 ± 1	*	37 ± 1 ^c^	32 ± 1 ^d^	62 ± 1 ^a^	51 ± 0 ^b^	19 ± 1	29 ± 0	*
Met	80 ± 7	n.d.		24 ± 0 ^a^	22 ± 1 ^a^	23 ± 1 ^a^	22 ± 1 ^a^	13 ± 0	12 ± 1	
Trp	85 ± 3	16 ± 0	*	274 ± 3 ^a^	240 ± 6 ^b^	137 ± 2 ^c^	127 ± 0.3 ^c^	141 ± 4	72 ± 2	*
Phe	195 ± 14	71 ± 1	*	34 ± 1 ^c^	34 ± 2 ^c^	48 ± 1 ^a^	39 ± 1 ^b^	20 ± 1	22 ± 4	
Ile	213 ± 8	45 ± 1	*	31 ± 0 ^c^	24 ± 1 ^d^	52 ± 2 ^a^	39 ± 1 ^b^	14 ± 0	22 ± 5	*
Leu	273 ± 15	35 ± 2	*	44 ± 1 ^c^	37 ± 0 ^d^	80 ± 1 ^a^	65 ± 1 ^b^	22 ± 0	37 ± 6	*
Lys	498 ± 7	57 ± 4	*	31 ± 0 ^d^	45 ± 2 ^c^	93 ± 2 ^a^	79 ± 3 ^b^	27 ± 3	45 ± 2	*

RP (whole-wheat raw pasta), CP (whole-wheat cooked pasta), RPA (whole-wheat raw pasta with algae), CPA (whole-wheat cooked pasta with algae), SPI (*Spirulina*), and HIM (*Himanthalia elongata*). dw, dry weight; fw, fresh weight; n.d., non-detected. Within each line, different letters represent significant differences between RP, CP, RAP, and CAP, at *p* < 0.05. Within each line, * represent significant differences between two different samples (i.e., CP and CAP expressed in fw or SPI and HIM), at *p* < 0.05.

## Data Availability

Data is contained within the article.
